# Transcriptomic evidence for an energetically advantageous relationship between *Syntrophomonas wolfei* and *Methanothrix soehngenii*

**DOI:** 10.1111/1758-2229.13276

**Published:** 2024-05-10

**Authors:** Maaike S. Besteman, Anna Doloman, Diana Z. Sousa

**Affiliations:** ^1^ Laboratory of Microbiology Wageningen University & Research Wageningen The Netherlands; ^2^ Centre for Living Technologies EWUU Alliance Utrecht The Netherlands

## Abstract

Syntrophic interactions are key in anaerobic food chains, facilitating the conversion of complex organic matter into methane. A typical example involves acetogenic bacteria converting fatty acids (e.g., butyrate and propionate), a process thermodynamically reliant on H_2_ consumption by microorganisms such as methanogens. While most studies focus on H_2_‐interspecies transfer between these groups, knowledge on acetate cross‐feeding in anaerobic systems is lacking. This study investigated butyrate oxidation by co‐cultures of *Syntrophomonas wolfei* and *Methanospirillum hungatei*, both with and without the addition of the acetate scavenger *Methanothrix soehngenii*. Growth and gene expression patterns of *S. wolfei* and *M. hungatei* were followed in the two conditions. Although butyrate consumption rates remained constant, genes in the butyrate degradation pathway of *S. wolfei* were less expressed in the presence of *M. soehngenii*, including genes involved in reverse electron transport. Higher expression of a type IV‐pili operon in *S. wolfei* hints to the potential for direct interspecies electron transfer between *S. wolfei* and *M. soehngenii* and an energetically advantageous relationship between the two microorganisms. Overall, the presence of the acetate scavenger *M. soehngenii* positively influenced the energy metabolism of *S. wolfei* and highlighted the relevance of including acetate scavengers when investigating syntrophic fatty acid degradation.

## Graphical abstract

We studied predefined co‐cultures to investigate the influence of acetate scavenger *Methanothrix soehngenii* on butyrate oxidation by a co‐culture of *Syntrophomonas wolfei* and *Methanospirillum hungatei*. RNAseq was used to compare the gene expression profiles of syntroph *S. wolfei* with and without the presence of the acetate scavenger *M. soehngenii*.

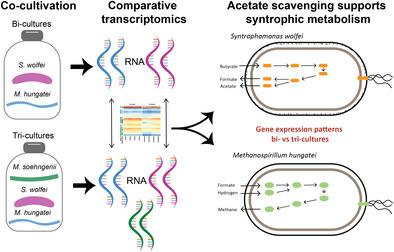

## INTRODUCTION

Syntrophy is a widespread microbial interspecies relationship, which is characterised by a thermodynamically constrained interdependency. One species, usually termed as the syntroph, can only convert certain substrates if the products of the reaction are maintained at a low concentration by a syntrophic partner (McInerney et al., 2009). This dynamic leads to cross‐feeding, exemplified in processes such as the conversion of fatty acids like butyrate. The anaerobic oxidation of butyrate to acetate and hydrogen by acetogenic bacteria (butyrate^−^ + 2H_2_O ➔ 2acetate^−^ + H^+^ + 2H_2_) has a positive Δ*G* ^0^′ of +48.1 kJ/reaction under standard conditions (Thauer et al., 1977). Only when the H_2_‐partial pressure in the environment is kept low, for example, by the activity of hydrogenotrophic methanogens, is the Δ*G*′ lowered, making the reaction thermodynamically feasible (Thauer et al., 1977). For a H_2_ partial pressure as low as 10^−6^ atm, the Δ*G*′ of the given reaction for butyrate oxidation becomes −20.4 kJ/reaction.

The conversion of fatty acids by syntrophic associations is well‐acknowledged in anaerobic digestion (AD), converting complex organic matter into a renewable energy in the form of biogas (Ferguson et al., 2016; Yadav et al., 2022). A stable cooperation between the syntrophic fatty acid oxidisers and their methanogenic partners is key to an efficient AD process (Mathai et al, 2020; Yue et al, 2021). These syntrophic interactions have mainly been investigated in bi‐cultures, pairing syntrophs with hydrogenotrophic (i.e., H_2_‐consuming) methanogens (Beaty & McInerney, 1989; Sieber, Le & Mcinerney, 2014). In these bi‐cultures, acetate is not used and accumulates in the medium. However, in natural anoxic environments, acetate is scavenged by, for example, acetoclastic methanogens. This can potentially be advantageous for energy conservation by the syntrophic bacteria, as a lower acetate concentration will favour thermodynamics of fatty‐acid oxidation. Using the example of butyrate oxidation, keeping acetate levels at 10 mM (and a $p_{H_{2}}$ of 10^−6^ atm) results in a Δ*G*′ of −43.2 kJ/reaction. A positive influence of decreased acetate concentration has been reported for the butyrate‐oxidising syntroph NSF‐2, which showed a higher butyrate conversion rate in the presence of *Methanosarcina barkeri* MS (Dwyer et al., 1988). However, the acetate concentration in that study was only slightly decreased by the methanogen, and no underlying mechanism for the improved substrate conversion rate was discussed. Therefore, despite the theoretical thermodynamic advantage of having an acetate scavenger in the system, further research is needed to understand its specific influence on the energy metabolism of syntrophic microorganisms.

In this study, we address the importance of acetate scavengers on the energy metabolism of *Syntrophomonas wolfei*. ‐ a butyrate degrader that was originally isolated in a co‐culture with hydrogenotrophic *methanogen Methanospirillum hungatei* (McInerney et al., 1981). Numerous studies have since focused on the bi‐culture of *S. wolfei* and *M. hungatei*, investigating the proteome and membrane complexes of both microorganisms (Crable et al., 2016; Schmidt et al., 2013; Sieber et al., 2015). Recently, the potential role of acyl‐protein modifications in regulating syntrophy in the bi‐culture was also researched (Fu et al., 2022). Together, these studies contribute a substantial body of knowledge on the metabolism of *S. wolfei*, making this organism an excellent candidate for investigating how it will be further influenced by the presence of an acetate scavenger, in addition to the H_2_‐scavenging methanogen. We studied bi‐ and tri‐cultures containing *S. wolfei* as a butyrate‐oxidising syntroph, *M. hungatei* as a hydrogenotrophic methanogen and *Methanothrix soehngenii* as an acetoclastic methanogen (only in tri‐cultures). We found that while butyrate consumption rates remained similar between bi‐ and tri‐cultures, *S. wolfei* in tri‐cultures had a decreased expression of genes related to both butyrate degradation and complexes involved in reverse electron transport. Additionally, we observed increased expression of a type IV‐pili operon of *S. wolfei*, which opens up the discussion for direct interspecies electron transfer (DIET) between *S. wolfei* and *M. soehngenii*. If occurring, this mechanism might be responsible for the lowering of the thermodynamic constraints on fatty acid oxidation by *S. wolfei*.

## EXPERIMENTAL PROCEDURES

### Source of microorganisms

*S. wolfei* (subsp. *wolfei*, DSM 102351^T^), *M. hungatei* JF1 (DSM 864^T^) and *M. soehngenii* GP6 (DSM 3671^T^) were obtained from the German Collection of Microorganisms and Cell Cultures (DSMZ, Braunschweig, Germany). *S. wolfei* was supplied in a co‐culture with *M. hungatei* JF1 as this microorganism is not available in an axenic culture.

### Co‐culture setup and cultivation medium

Bi‐ and tri‐cultures were established to investigate the influence of acetoclastic methanogens on syntrophic growth. These co‐cultures comprised *S. wolfei* as a butyrate‐degrading syntroph, *M. hungatei* as a hydrogenotrophic methanogen and in tri‐cultures, *M. soehngenii* as an acetoclastic methanogen. We grew four bi‐cultures and four tri‐cultures for RNA extraction. Additional bi‐ and tri‐cultures (*N* = 5 and *N* = 6, respectively) were grown to monitor growth and metabolites over time. All co‐cultures were grown anaerobically and non‐shaking at 37°C in 250 mL serum bottles including 100 mL N_2_‐flushed bicarbonate buffered medium as previously described (Stams et al., 1993). Bi‐cultures were started by a 10% v/v transfer of a growing bi‐culture in the late exponential phase into a fresh medium. Tri‐cultures were started by inoculating 10% v/v of the same bi‐culture into a grown pure culture of *M. soehngenii*, which had (nearly) finished consumption of 40 mM Na‐acetate. Bi‐ and tri‐cultures were grown with an 80:20 (v/v) N_2_:CO_2_ headspace at 1.5 bar containing 20 mM sodium butyrate as a sole carbon source. Pure cultures of *M. soehngenii* were grown with an 80:20 (v/v) N_2_:CO_2_ headspace at 1.5 bar containing 40 mM sodium acetate.

### Analytical techniques

Butyrate and other volatile fatty acids (acetate and formate) were measured using high‐performance liquid chromatography (HPLC). Liquid samples (0.5 mL) were taken daily from active co‐cultures, centrifuged and the supernatant was mixed with equal volume of 0.1 N sulphuric acid. Samples were run on a Shimadzu LC2030‐plus HPLC (Kyoto, Japan) equipped with a Shodex SH1821 column (Shodex, Japan) set at 45°C using 0.01 N sulphuric acid as eluent with a flow rate of 1 mL min^−1^. Peak detection was done using a refractive index detector Shimadzu RID‐20A (Shimadzu, Kyoto, Japan).

Methane was monitored using gas chromatography by injecting 0.2 mL of the (co)cultures headspace on a CompactGC^4.0^ (Interscience, Breda, The Netherlands) equipped with a 3 m × 0.32 mm Carboxen 1010 pre‐column (Sigma Aldrich, Germany), a 30 m × 0.32 mm Molsieve 5A column (Agilent, Santa Clara, CA) and a thermal conductivity detector (Interscience, Breda, The Netherlands). Injector, column and detector temperatures were 100, 140 and 110°C, respectively, with Argon as a carrier gas at a flowrate of 1 mL min^−1^.

### RNA extraction

Quadruplicate bi‐ and tri‐cultures were sampled for RNA extraction at the growth phase matching substrate consumption of 10 mM sodium butyrate. Biomass was harvested by centrifugation for 15 min at 10,000 g and 4°C (Sorvall Legend XTR, Thermo Fisher Scientific, Waltham, MA) and washed with pre‐cooled filter‐sterilised Tris‐EDTA (TE) buffer (10 mM Tris–HCl and 1 mM Ethylenediaminetetraacetic acid [EDTA], pH 8). Washed biomass was collected by centrifugation for 10 min at 4700 g and 4°C (Sorvall Legend XTR, Thermo Fisher Scientific, Waltham, MA), after which the pellets were snap‐frozen with liquid nitrogen and stored at −70°C until extraction was continued. Solutions of the MasterPure™ Gram‐positive DNA Purification kit (BioSearch Technologies, UK) were used in subsequent steps. Pellets were resuspended in 150 μL TE buffer and 1 μL of lysozyme solution was added, followed by incubation for 20 min at 37°C. Four microlitres of β‐mercaptoethanol were added and the cells were sonicated (Bendelin SONOPULS HD 3200 ultrasonic homogeniser) for 6 cycles of 20 s pulse and 30 s pause. A mixture of 150 μL of Gram‐positive lysis solution and 1 μL of proteinase K (50 μg/μL) was prepared at 37°C and added to the samples. The samples were incubated at 60°C for 15 min, interfered with vortexing every 5 min. After cooling on ice for 5 min, 175 μL of MPC protein precipitation reagent was added and samples were vortexed for 15 s. Cell debris was pelleted down by centrifugation for 10 min at 4700 g and 4°C (Microcentrifuge 5425‐R, Eppendorf, Germany) and the supernatant was collected. Finally, 400 μL of supernatant was used to complete RNA extraction using the Maxwell® RSC SimplyRNA cells kit (Promega, Madison, WI). The amount and purity of the isolated RNA was measured in a NanoDrop spectrophotometer, and the samples were stored at −70°C until sequencing.

### RNA sequencing and analysis

RNA samples were sequenced on the Illumina Novoseq 6000 platform (Novogene, UK) with a prokaryotic strand‐specific transcriptome library yielding paired‐end reads of 150 bp. Obtained sequences were trimmed by bbduk.sh of BBmap (v38.84) (ktrim = r k = 23 mink = 7 hdist = 1 tpe tbo qtrim = rl trimq = 30 ftm = 5 maq = 20 minlen = 50 hdist = 1), followed by fastQC quality check (v0.11.9). Three reference genomes were annotated with prokka (Seemann, 2014): *S. wolfei* (GenBank CP000448.1), *M. hungatei* (GenBank CP000254.1) and *M. soehngenii* (GenBank CP002565), producing the prokka fnn‐file output. Sequences were then mapped using bbsplit.sh against the fnn‐files of the three reference genomes. Mapped genes were counted using samtools view (version 1.10) (‐SF 260, cut‐f 3) and further analysed in Rstudio (v4.0.2), separately for each organism. To determine gene expression ranking within each of the two conditions, counts were normalised to transcripts per million. The bioconductor package DESeq2 (v1.30.16) was used for differential expression analysis (Love et al., 2014). To determine significant differential expression, we considered a fold change ≥1.5, and a *p*‐value adjusted by the Benjamini and Hochberg method ≤0.05 (Benjamini & Hochberg, 1995). Data visualisation was also performed in R, using DESeq2 normalised counts. DESeq2 normalised counts are included in Supporting Information S1. Raw data were deposited to the European Nucleotide Archive at project PRJEB64144 with study accession ERA24700593.

## RESULTS AND DISCUSSION

### *Growth of* S. wolfei *and* M. hungatei *co‐cultures with and without* M. soehngenii

This study set out to investigate the importance of acetate scavenging on the metabolism of the syntrophic butyrate degrader *S. wolfei*. Bi‐cultures containing *S. wolfei* as a butyrate degrader and *M. hungatei* as a hydrogenotrophic methanogen were compared with the tri‐cultures, which, in addition to *S. wolfei* and *M. hungatei,* also had *M. soehngenii* as an acetoclastic methanogen.

**FIGURE 1:**
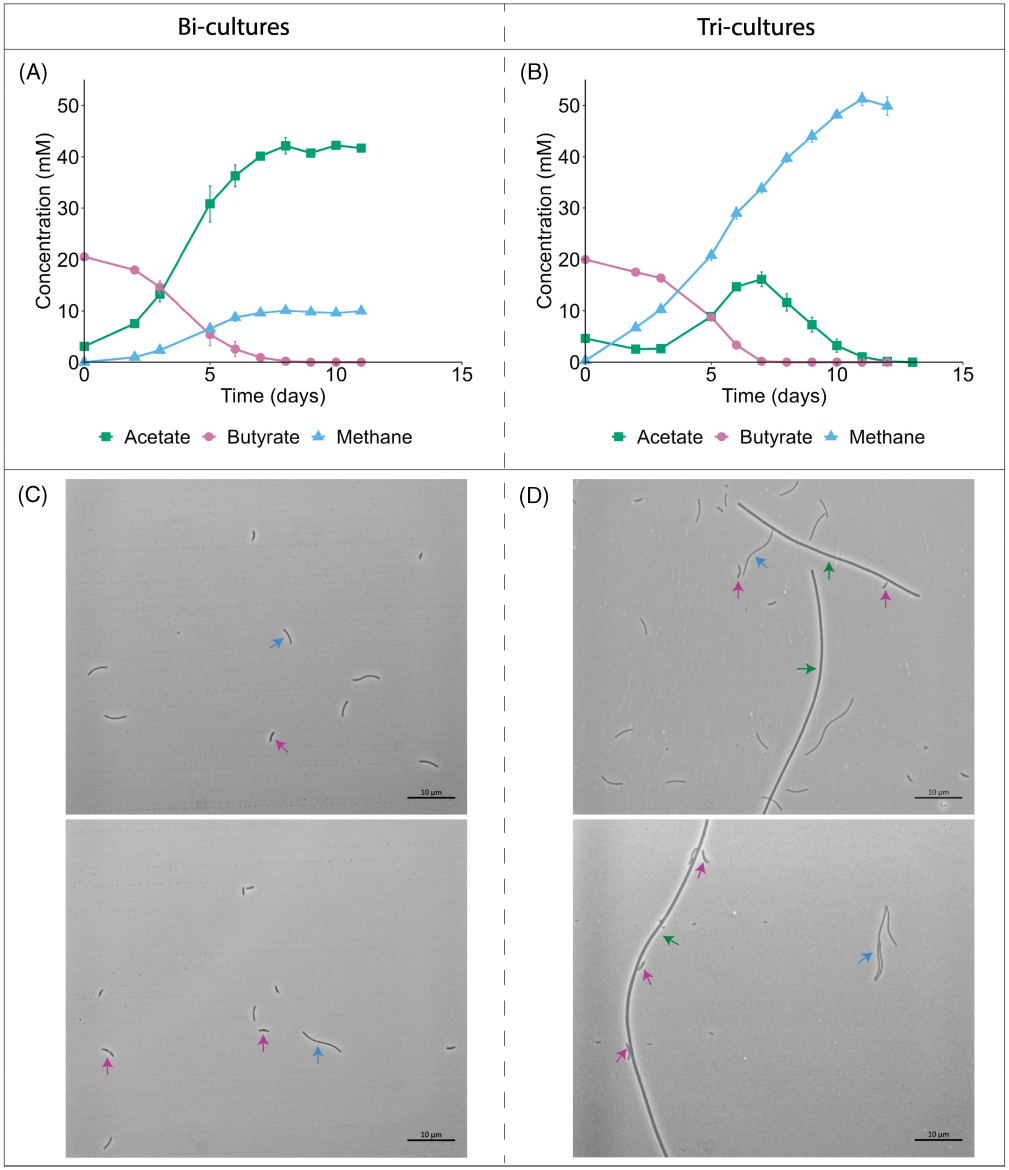
Growth curves (A, B) and phase‐contrast microscopy (C, D) of bi‐cultures of *Syntrophomonas wolfei* and *Methanospirillum hungatei* and tri‐cultures of *S. wolfei*, *M. hungatei* and an acetate scavenger *Methanothrix soehngenii*. (A, B) Error bars represent standard deviation over bi‐culture replicates (*n* = 5) and tri‐cultures replicates (*n* = 6). Hydrogen levels are not reported as they remained below detection limit (≤300 ppm). (C, D) Coloured arrows indicate the three microorganisms with distinct morphologies: pink arrows for *S. wolfei*, blue arrows for *M. hungatei* and green arrows for *M. soehngenii*.

Our initial hypothesis posited that the decreased acetate concentration in tri‐cultures could amplify the available energy derived from butyrate oxidation due to a higher thermodynamic driving force. This change was expected to accelerate butyrate consumption rates. We observed that while the addition of acetate scavenger *M. soehngenii* to tri‐cultures successfully limited acetate build‐up (Figure 1A,B), the rates of butyrate oxidation remained similar: 5.0 ± 0.6 mM/day for bi‐cultures and 5.5 ± 0.4 mM/day for tri‐cultures. This shows that the potential benefit for *S. wolfei* in having *M. soehngenii* is not reflected in butyrate consumption rate. However, we observed that the addition of *M. soehngenii* has always resulted in stable growing tri‐cultures. By contrast, transfers of bi‐cultures were often subject to extended lag‐phases or lacked growth completely (data not included). With phase‐contrast microscopy of bi‐ and tri‐cultures (Figure 1C,D) we observed that *S. wolfei* in tri‐cultures was often found in close proximity to *M. soehngenii*. These observations suggest that the benefit of the presence of *M. soehngenii* likely lies elsewhere than improving the syntrophs' substrate consumption rates.

### *Gene expression analysis of* S. wolfei *and* M. hungatei *in co‐cultures with and without* M. soehngenii

To understand the impact of the acetate scavenger *M. soehngenii* on the metabolism of the syntrophic bacterium *S. wolfei*, we conducted comparative transcriptomics. Bi‐ and tri‐cultures were sampled for RNA extraction at time points when equal amounts of butyrate were consumed (10 mM), while acetate concentrations were 7.3 ± 2.5 mM and 21.9 ± 1.2 mM in the bi/tri‐cultures, respectively. The differential gene expression analysis of *M. hungatei* in bi‐ versus tri‐cultures showed 898 significant DE genes (fold change ≥1.5, adjusted *p*‐value ≤0.05), with 516 and 382 genes being up‐ and downregulated in tri‐cultures, respectively (Figure S2 and Supporting Information S1). *S. wolfei* gene expression in bi‐ versus tri‐cultures revealed significant differential expression for a total of 424 genes, with 201 and 222 genes up‐ and downregulated in tri‐cultures, respectively (Figure S2 and Supporting Information S1). The variance in gene expression profiles of *S. wolfei* and *M. hungatei* were visualised with a principal component analysis (Figure S1). Clusters of orthologous groups were assigned to all significant DE genes (Figures S3 and S4). Following the focus point of this study, we looked into the differential expression of genes specifically associated with carbon/energy metabolism and reverse electron transport. However, we also noted that the presence of acetate scavenger *M. soehngenii* resulted in an interesting expression pattern of genes related to chemotaxis and cell motility, which we also discuss here.

### *Gene expression of* M. hungatei *in bi‐ versus tri‐cultures*

**FIGURE 2:**
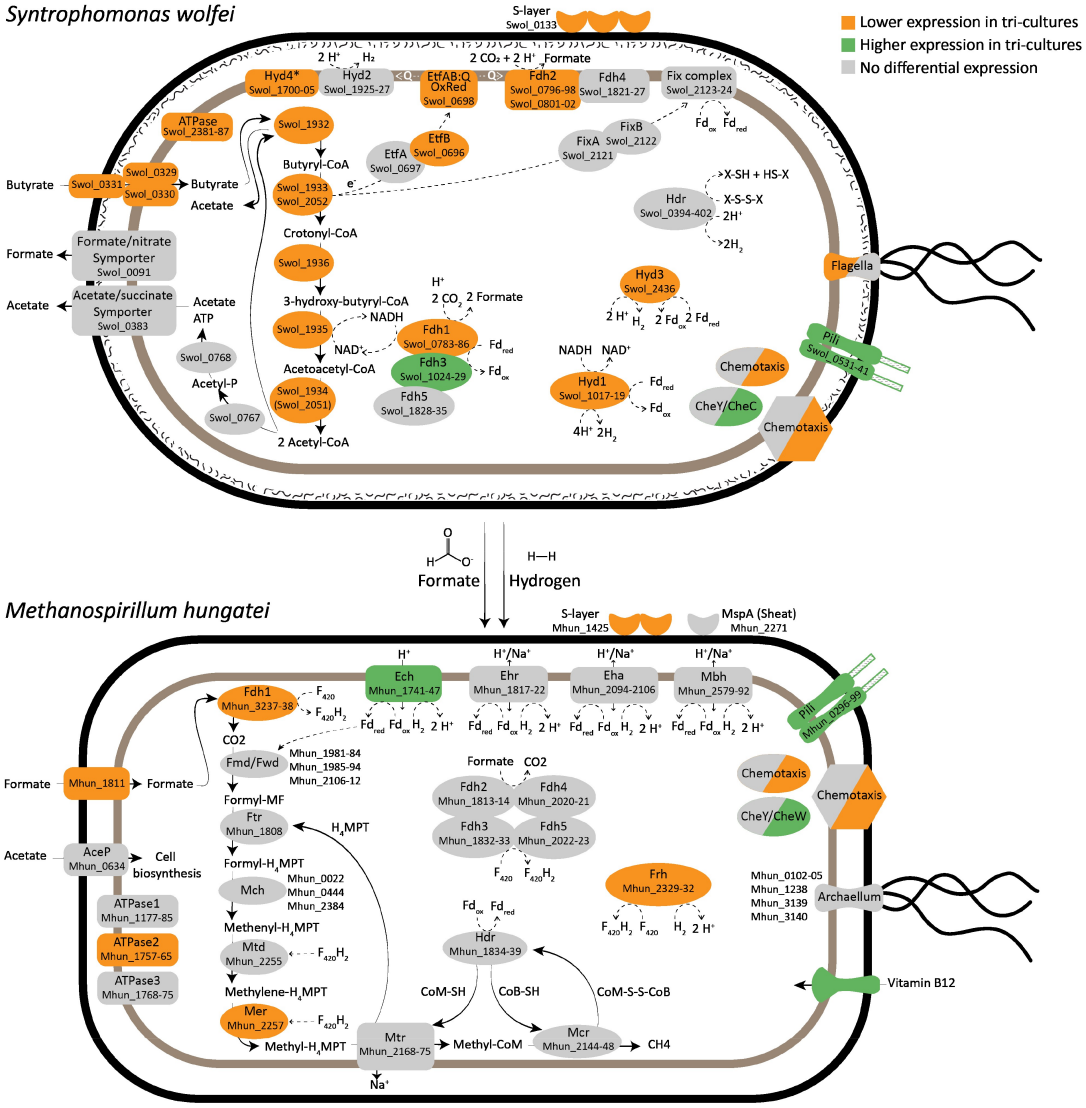
Metabolic models of *Syntrophomonas wolfei* and *Methanospirillum hungatei* based on RNAseq results illustrating the influence of the presence of acetate scavenger *Methanothrix soehngenii* on their main metabolism. Orange boxes indicate lower expression in tri‐cultures compared to bi‐cultures. Green boxes indicate higher expression in tri‐cultures and grey boxes indicate no significant differential expression. Solid arrows indicate the flow of compounds. Dashed arrows indicate the flow of electrons. Etf, electron transfer flavoprotein; Fdh, formate dehydrogenase; Fmd/Fwd, formyl‐methanofuran dehydrogenase; Ftr, formyl MFR:tetrahydromethanopterin formyl transferase; Hdr, heterodisulphide reductase; Hyd, hydrogenases (Ech, Ehr, Eha, Frh and Mbh); Mch, methenyl‐H_4_MPT tetrahydromethanopterin cyclohydrolyase; Mcr, methyl‐CoM reductase; Mer, methylene‐H_4_MPT reductase; Mtd, methylene‐H_4_MPT dehydrogenase; Mtr, H_4_MPT S‐methyltransferase. *Hyd4 is not mentioned in previous genomic and gene/protein expression studies of *S. wolfei*. This study is the first to report this operon as a potential membrane‐bound NADH:ubiquinone oxidoreductase.

Differential expression in the central metabolism of *M. hungatei* is illustrated alongside with *S. wolfei* (Figure 2). Most gene complexes involved in the central metabolism of *M. hungatei* are less expressed in the tri‐cultures. Interestingly, one Ech hydrogenase (Mhun_1741‐47) had a significantly higher expression in tri‐culture condition (average DESeq2 normalised counts of 726 and 1651 in bi‐ vs. tri‐cultures, respectively). This membrane‐bound Ech hydrogenase couples oxidation of H_2_ to reduction of ferredoxin making use of the proton motive force (pmf). The higher expression of this protein complex in tri‐cultures suggests a higher requirement for generating reduced ferredoxin, used for the reduction of CO_2_ to formyl‐methanofuran or for the oxidation of H_2_.

Only a few genes related to chemotaxis in *M. hungatei* were significantly differentially expressed, with mainly lower expression in the tri‐culture condition (Figure S8). Exceptions were one CheY‐like protein (Mhun_0315) and one CheW protein (Mhun_0898), with overall low expression but significant higher expression in tri‐cultures. What stood out in the gene expression profile of *M. hungatei*, was a significantly higher expression in tri‐cultures of a gene cluster suggested to be archaeal‐type pili (Mhun_0296‐99, Figures 2 and S8) (Gunsalus et al., 2016). This gene cluster is distinct from the previously studied electrically conductive archaellum of *M. hungatei* (Mhun_3140), suggested to be involved in DIET (Poweleit et al., 2016; Walker et al., 2019). The hypothesised archaeal pili cluster Mhun_0296‐99 is orthologous to pili of archaeon *Haloferax volcanii*, in which it is suggested to play a role in cellular adhesion or communication. It is likely they have a similar role in our co‐cultures, with more involvement in the tri‐culture condition. Unfortunately, the current lack of knowledge on the exact role of *M. hungatei* archaeal pili hampers a definite hypothesis on their function.

### *Gene expression of* S. wolfei *in bi‐ versus tri‐cultures*

#### *Carbon and energy metabolism of* S. wolfei

Although the butyrate conversion rates of *S. wolfei* were similar in bi‐ and tri‐culture conditions, genes involved in the carbon and energy metabolism of *S. wolfei* were differentially expressed (Figure 2). Starting from the import of butyrate by the tripartite ATP‐independent periplasmic transporter (TRAP transporter, Swol_0329‐31) up to its conversion into two acetyl‐CoA, all genes involved were significantly less expressed in tri‐culture conditions (Figures 2 and S5). Interestingly, genes finalising the pathway by conversion of acetyl‐CoA to acetate and ATP (Swol_0767‐0768) and the export of acetate (Swol_0383) were not differentially expressed. These results show that *S. wolfei* in tri‐cultures was able to degrade butyrate with similar rates as in bi‐cultures, but with fewer transcripts of the genes in the main pathway.

Export of acetate from *S. wolfei* cells is proposed to occur via a succinate‐acetate symporter SatP (Swol_0383), which could contribute to the build‐up of PMF (Crable et al., 2016) similar to what has been suggested for formate transport in *Pelotomaculum schinkii* (Hidalgo‐Ahumada et al., 2018). However, although Swol_0383 was highly expressed in both conditions, it was not differentially expressed (DESeq2 normalised counts of 3400 and 3500 in bi‐ and tri‐cultures, respectively). The alternative, diffusion of acetate across the cell membrane in the form of acetic acid, is unlikely, given that acetic acid has a p*K*_a_ of 4.76 and internal pH of *S. wolfei* is expected to be around 7 (Slonczewski et al., 2009).

#### *Reverse electron transport in* S. wolfei

**FIGURE 3:**
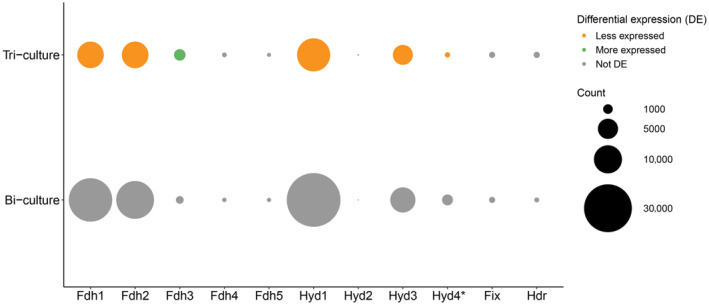
Average expression of gene clusters involved in reverse electron transport in *Syntrophomonas wolfei*. DESeq2 normalised counts of all genes in the operon are averaged. Colours depict whether the gene was upregulated (green) or downregulated (orange) compared to the bi‐culture condition. *Hyd4 is not mentioned in previous genomic and gene/protein expression studies of *S. wolfei*. This study is the first to report this operon as a potential membrane‐bound NADH:ubiquinone oxidoreductase.

The β‐oxidation pathway of *S. wolfei* degrading butyrate includes two oxidation steps from which electrons need to be transferred to a suitable electron acceptor. In this role, *S. wolfei* employs five formate dehydrogenases (Fdh), three hydrogenases (Hyd), a fix‐complex and a heterodisulphide reductase (Hdr) (Figures 3 and S6). The specific complex preferred for electron transport has been shown to be influenced by growth conditions and the presence of a syntrophic partner (Crable et al., 2016; Sieber et al., 2015). The average expression of the complexes in this study is illustrated (Figure 3).

In the first oxidation step by butyryl‐CoA dehydrogenase (Swol_1933 or Swol_2052), electrons are transferred to membrane‐bound complexes via an electron‐transferring flavoprotein complex (EtfAB, gene products of Swol_0696‐97, or FixAB, gene products of Swol_2121‐22). Only the expression of EtfB was significantly lower in tri‐cultures (Figures 3 and S6). To overcome a redox‐unfavourable transfer of the electrons, EtfAB first transfers electrons to an EtfAB:menaquinone oxidoreductase (Swol_0698), which reduces menaquinone to menaquinol. Swol_0968 was significantly less expressed in tri‐cultures (Figures 3 and S6). From the menaquinol‐pool, electrons can be transferred to two types of membrane‐bound complexes: (1) Hydrogenase complexes 2 or 4 (Hyd2 and Hyd4) to produce hydrogen and (2) Formate dehydrogenase complexes 2 or 4 (Fdh2 and Fdh4) to produce formate. Fdh2, product of Swol_0796‐802, was in both conditions the main expressed membrane‐bound reducing complex (DESeq2 normalised counts of 18,471 and 8946, in bi‐ and tri‐cultures, respectively) and was significantly less expressed in tri‐cultures (Figures 3 and S6). Previous proteomics studies described membrane‐bound Hyd2 (gene product of Swol_1925‐27) as a highly expressed hydrogenase important for electron transport to hydrogen (Crable et al., 2016). Hyd2 had, however, very low expression in both bi‐ and tri‐cultures of this study (DESeq2 normalised counts of 15 and 13, respectively). These low counts suggest that the activity of Hyd2 was not relevant for reverse electron transport during the growth of our co‐cultures.

We identified a second potential membrane‐bound hydrogenase that has not been reported in any previous studies: gene product of Swol_1700‐1705, here named Hyd4. Based on previous genome annotations (RefSeq NC_008346.1 and GenBank CP000448.1), our annotation with Uniprot BLAST and Interpro‐scan, Hyd4 predicts to be membrane bound and have NADH‐quinone oxidoreductase activity. The operon consists of one membrane‐spanning 2Fe‐2S iron–sulphur cluster‐binding protein (Swol_1700), and non‐membrane‐bound proteins associated with NAD(P)‐reducing hydrogenase subunits. Based on these annotations and the membrane‐spanning sub‐unit, hydrogenase activity is likely. Unfortunately, the operon has not been reported in previous studies on *S. wolfei* investigating either its complete genome (Sieber et al., 2010), its full proteome (Sieber et al., 2015), active membrane‐associated complexes (Crable et al., 2016), or even specifically NADH reductases (Losey et al., 2017; Müller et al., 2009). It remains unclear why this operon has been overlooked in previous studies and what its exact role is in reverse electron transport in *S. wolfei*.

Overall, the expression of all membrane‐bound complexes suggests that electrons of the menaquinol pool are in both bi‐ and tri‐culture conditions mainly transferred to the Fdh2 complex to produce formate. The high gene expression of Fdh2 agrees with data from a previous study showing strong expression of the complex on a proteome level (Schmidt et al., 2013). Unfortunately, this hypothesis could not be substantiated by measuring formate production, due to the scavenging of both hydrogen and formate by *M. hungatei*. What supports our hypothesis is that it has been previously suggested that formate may play a more important role than hydrogen for the syntrophic organisms *Syntrophobacter fumaroxidens* and *P. schinkii* (De Bok et al., 2002; Hidalgo‐Ahumada et al., 2018). An earlier study investigating whole granules, however, showed that electron transfer mainly occurred through hydrogen and considered formate to be not of major importance for interspecies electron transfer (Schmidt & Ahring, 1995).

The membrane‐bound complexes involved in reverse electron transport require a PMF, as protons in the periplasm are needed for the reduction to hydrogen or formate. *S. wolfei* does not have the capability to create a pmf via a respiratory system. Instead, an ATPase complex (Swol_2381‐88) is suggested to create a pmf by hydrolysation of ATP (Sieber et al., 2015). The generated pmf can be used by Hyd2, Hyd4, Fdh2 and Fdh2 for the reoxidation of menaquinol and the production of hydrogen or formate. The ATPase was significantly less expressed in tri‐cultures (Figures 3 and S5). This suggests that there was less need for *S. wolfei* tri‐cultures to have pmf generation by hydrolysation of ATP, potentially leaving a bigger pool of ATP left for other bioprocesses.

A second approach *S. wolfei* could use to ensure maintenance of a pmf is to make use of its cell wall. It is proposed that the cell wall of *S. wolfei*, among which the S‐layer (Swol_0133), plays a role in maintaining its pmf and thereby promotes the formation of formate in the periplasm, for which the pmf is essential (Schmidt et al., 2013). The highest expressed gene in both bi‐ and tri‐cultures was Swol_0133 (DESeq2 normalised counts of 58.6 × 10^4^ and 28.3 × 10^4^, respectively). Swol_0133 is consistently annotated as a hypothetical protein but is predicted to encode for a surface‐layer protein (Schmidt et al., 2013). It is significantly less expressed in tri‐cultures (Figures 3 and S5), which could suggest that promoting periplasmic formate production is less needed since *S. wolfei* could be more efficient in its electron transfer mechanisms.

The second oxidation step in the β‐oxidation pathway couples the reduction of NAD^+^ to the oxidation of 3‐hydroxy‐butyryl‐CoA to acetoacetyl‐CoA. NADH can be re‐oxidised by the soluble complexes Hyd1, Fdh1, Fdh3 or Fdh5. In both bi‐ and tri‐cultures, Hyd1 (Swol_1017‐19) is the highest expressed soluble hydrogenase (DESeq2 normalised counts of 3.8 × 10^4^ and 1.4 × 10^4^, respectively) and Fdh1 (Swol_0783‐66) is the highest expressed soluble formate dehydrogenase (DESeq2 normalised counts of 2.5 × 10^4^ and 8.9 × 10^3^, respectively). Both complexes Hyd1 and Fdh1 are significantly less expressed in the tri‐cultures (Figures 3 and S6). Interestingly, Fdh3 (Swol1024‐29) is expressed in addition to Fdh1 in bi‐ and tri‐culture conditions (DESeq2 normalised counts of 602 and 1487, respectively), but with significantly higher expression in the tri‐cultures (Figures 3 and S6). This makes Fdh3 the only complex involved in reverse electron transport to hydrogen or formate with higher expression in tri‐cultures. It remains unclear why the presence of *M. soehngenii* led to this shift and why this contrast in DE direction was observed.

#### *Chemotaxis and motility in* S. wolfei

Multiple genes integral to chemotaxis or motility showed significant differential expression between bi‐ and tri‐culture conditions (Figure S7). Main differences revolved around a lower expression of genes associated with chemotaxis or flagella production in tri‐culture condition. Among these, Swol_0198, annotated as flagellin and a related hook‐associated protein, exhibited significantly lower expression in tri‐cultures compared to bi‐cultures (DESeq2 normalised counts of 32.9 × 10^4^ and 19.6 × 10^4^ in bi‐ and tri‐cultures, respectively). Two other gene clusters involved in flagella production, Swol_0233‐35 and Swol_0847‐50, also showed lower expression in tri‐cultures. Additionally, two operons involved in chemotaxis, Swol_1446‐1451 and Swol_1453‐57, had a lower expression in tri‐cultures. This overall decrease in expression of genes involved in motility and chemotaxis suggests a lower need for movement towards syntrophic partner, likely due to a smoother electron scavenging in the more stable butyrate converting tri‐cultures.

Interestingly, an operon involved in type IV‐pili production (Swol_0531‐41) showed significantly higher expression in tri‐cultures with *M. soehngenii*. Type IV‐pili have been implicated in DIET in *Syntrophus aciditrophicus* (Walker et al., 2020). *Methanothrix* has also been suggested to be a potential DIET partner to *Syntrophomonas* in Fe_3_O_4_‐enriched digesters and to *Geobacter* species in wastewater aggregates (Rotaru et al., 2013; Zhao et al., 2018). The higher expression of Swol_0531‐41 in *S. wolfei* suggests its potential role in DIET between *S. wolfei* and *M. soehngenii*, likely reducing the partners' dependence on hydrogenase complexes for electron transfer. The lower burden to keep up with reverse electron transport could be the reason for a lower expression of the β‐oxidation pathway and the hydrogenase complexes, an aspect that deserves future research.

## CONCLUSION

The addition of acetate scavenger *M. soehngenii* to syntrophic butyrate‐oxidising tri‐cultures successfully limited acetate build‐up compared to the bi‐cultures containing only *S. wolfei* and *M. hungatei*. Although the kinetics of butyrate conversion were not affected by the presence of the acetoclastic methanogen, transcriptomic results show that it influenced the expression of the syntrophic energy metabolism. Complexes directly involved in the β‐oxidation pathway, as well as those critical for facilitating reverse electron transport from this pathway, were less expressed in tri‐culture condition. This suggests a potential lower thermodynamic pressure on *S. wolfei* to maintain a low redox pool. Besides complexes producing hydrogen or formate, pili of *S. wolfei* were significantly higher expressed in tri‐cultures, which is a potential route for DIET. Our study thereby supports the previous suggestion that DIET can occur between *Syntrophomonas* and *Methanothrix* (previously *Methanosaeta*). The additional high expression of archaeal‐type pili of *M. hungatei* underlines the influence that the presence of acetate scavenger *M. soehngenii* could have on routes of interspecies electron transfer, essential for the community to maintain proper volatile fatty acid degradation. Overall, this study highlights the relevance of incorporating acetate scavenging when investigating syntrophic growth performance in defined co‐cultures. Our results contribute to a better understanding of syntrophic metabolisms in AD systems, crucial for understanding microbial interactions in natural environments and for ensuring a stable biogas‐producing microbial community in engineered AD systems.

## AUTHOR CONTRIBUTIONS

**Maaike S. Besteman:** Conceptualization (equal); investigation (lead); methodology (lead); validation (lead); visualization (lead); writing – original draft (lead). **Anna Doloman:** Conceptualization (equal); investigation (supporting); methodology (equal); supervision (equal); writing – review and editing (lead). **Diana Z. Sousa:** Conceptualization (equal); funding acquisition (lead); methodology (equal); supervision (equal); writing – review and editing (lead).

## CONFLICT OF INTEREST STATEMENT

The authors have no conflict of interest to disclose.

## Supporting information


**Data S1.** DEseq2 differential expression data.

**Data S2.** Supporting Information.

## Data Availability

Raw data has been deposited to the European Nucleotide Archive at project PRJEB64144 with study accession ERA24700593.
