# Supplementary material for: Transcriptomic evidence for an energetically advantageous relationship between Syntrophomonas wolfei and Methanothrix soehngenii
Source: Environ Microbiol Rep. 2024 May 10;16(3):e13276. doi: 10.1111/1758-2229.13276 (PMC11087674; doi:10.1111/1758-2229.13276)
Supplement: Supplementary file 2 — Data S2. Supporting Information. [file EMI4-16-e13276-s002.docx]

**SUPPLEMENTARY FILE 1**


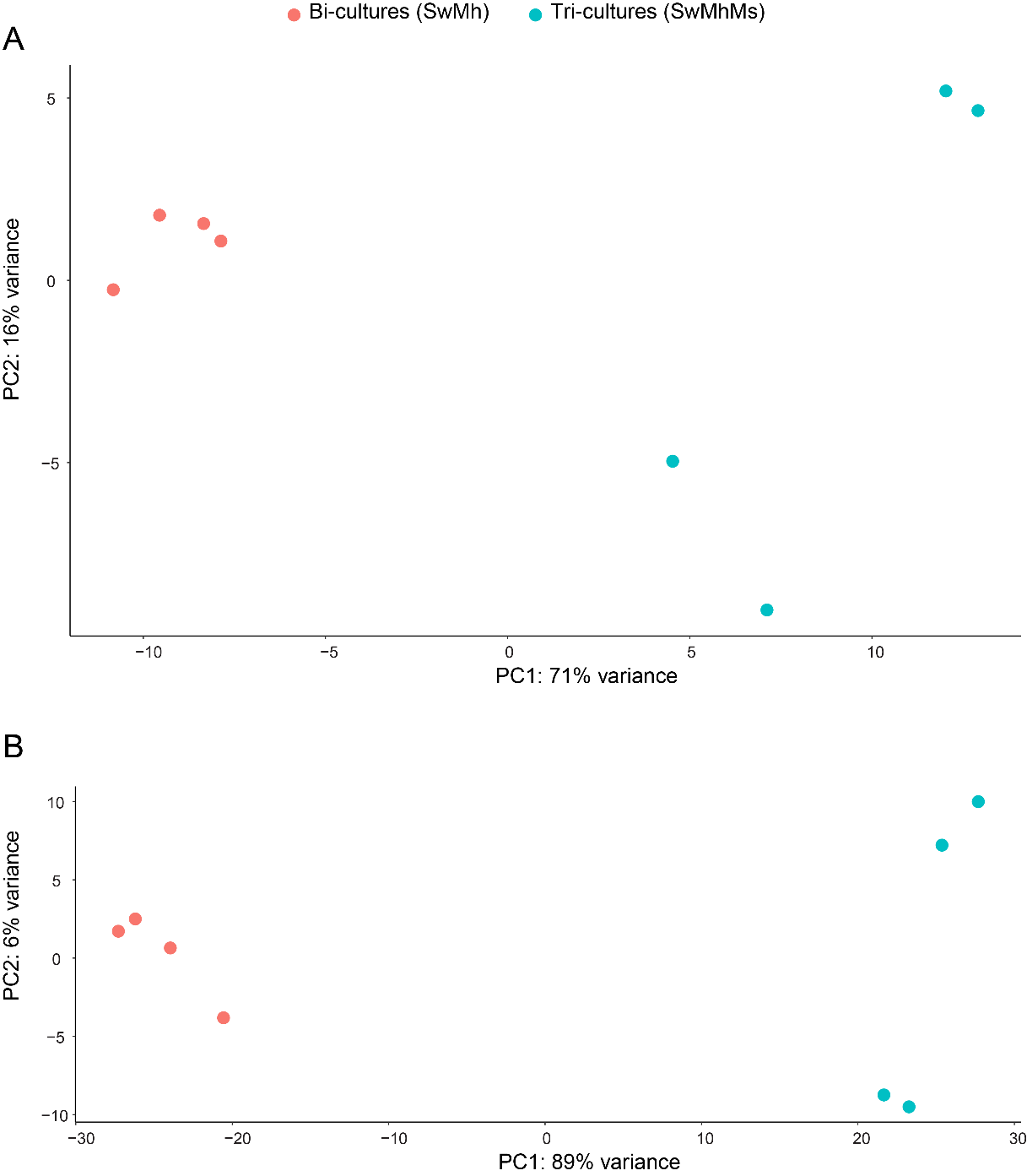


**Figure S1 |** Principal component analysis of DESeq2 normalized counts of (A) *S. wolfei* and (B) *M. hungatei* in bi- and tri-cultures.


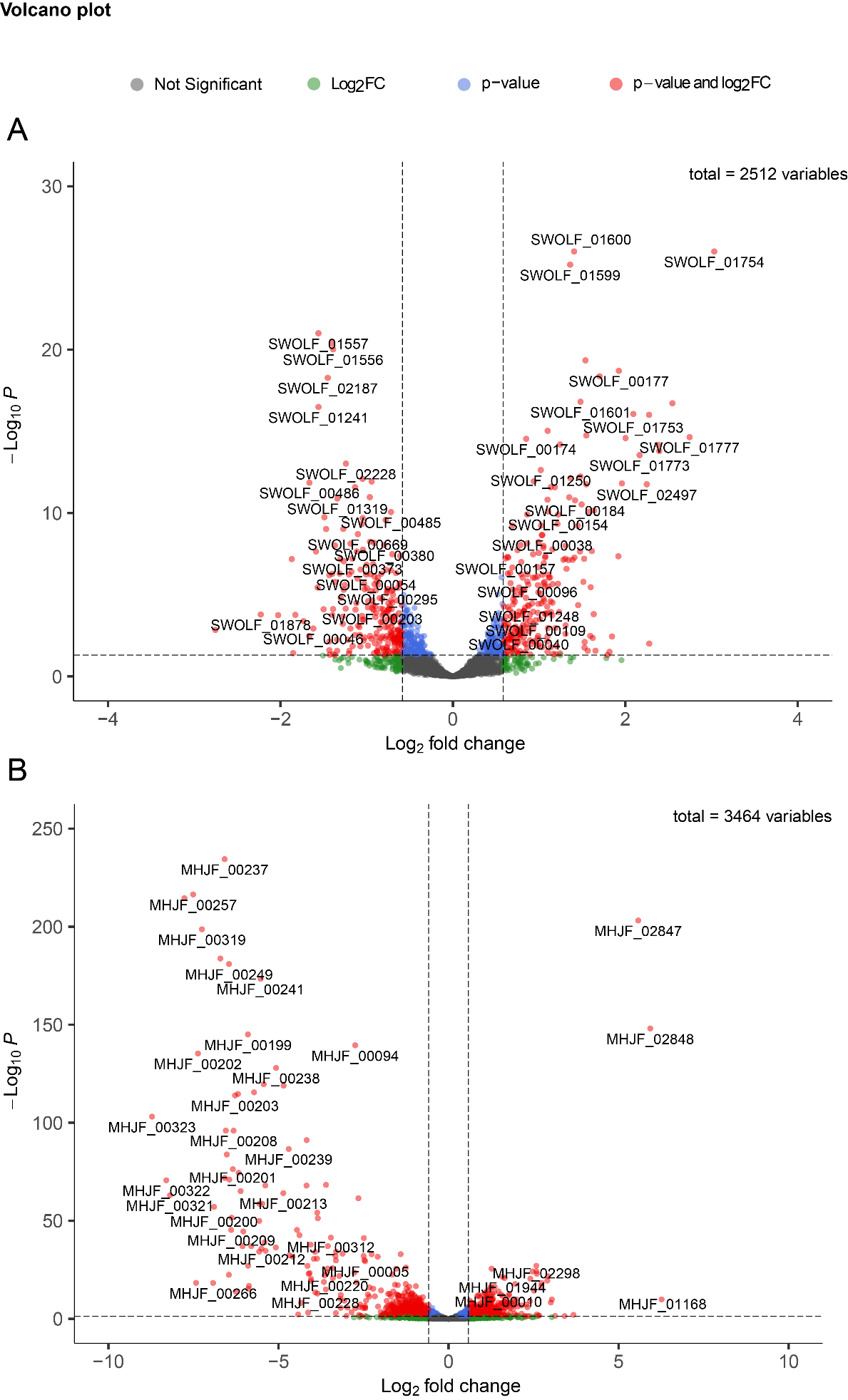


**Figure S2 |** Volcano plot showing differential expression of *S. wolfei* (A) and *M. hungatei* (B). Negative log2-fold change (log2-FC) indicates higher expression in tri-cultures, positive log2-FC indicates lower expression in tri-cultures. Dashed lines show a fold change (FC) cut-off of 1.5 and adjusted P-value cutoff of 0.05.

**Figure S3 |** **Counts of Clusters of Orthologous Groups (COG) assigned to all differentially expressed (DE) genes of *S. wolfei*.** COG categories of all DE genes of *S. wolfei* down-regulated (orange bars) or upregulated (green bars) in tri-cultures compared to bi-culture condition. COG categories were assigned for all significant differentially expressed genes (fold change ≥ 1.5, adjusted p-value ≤ 0.05) using the public online resource EggNOG-mapper (version 6.0, <http://eggnog-mapper.embl.de> )

**Figure S4 | Counts of Clusters of Orthologous Groups (COG) assigned to all genes of *M. hungatei* differentially expressed (DE).** COG categories of all DE genes of *M. hungatei* down-regulated (orange bars) or upregulated (green bars) in tri-cultures compared to bi-culture condition. COG categories were assigned for all significant differentially expressed genes (fold change ≥ 1.5, adjusted p-value ≤ 0.05) using the public online resource EggNOG-mapper (version 6.0, <http://eggnog-mapper.embl.de>)


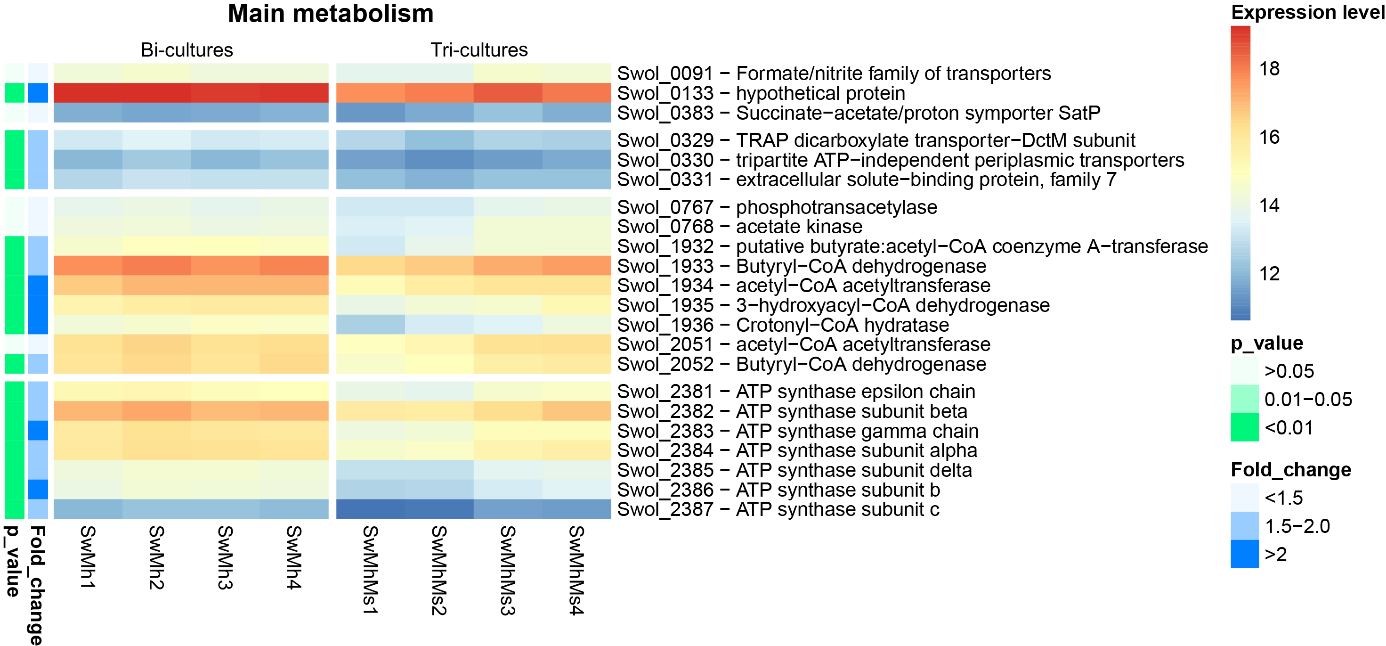


**Figure S5 |** Differential expression of *S. wolfei* genes involved in the main metabolism for butyrate degradation in bi- and tri-cultures. Variance stabilizing transformation on DESeq2 normalized counts was applied for visualization.


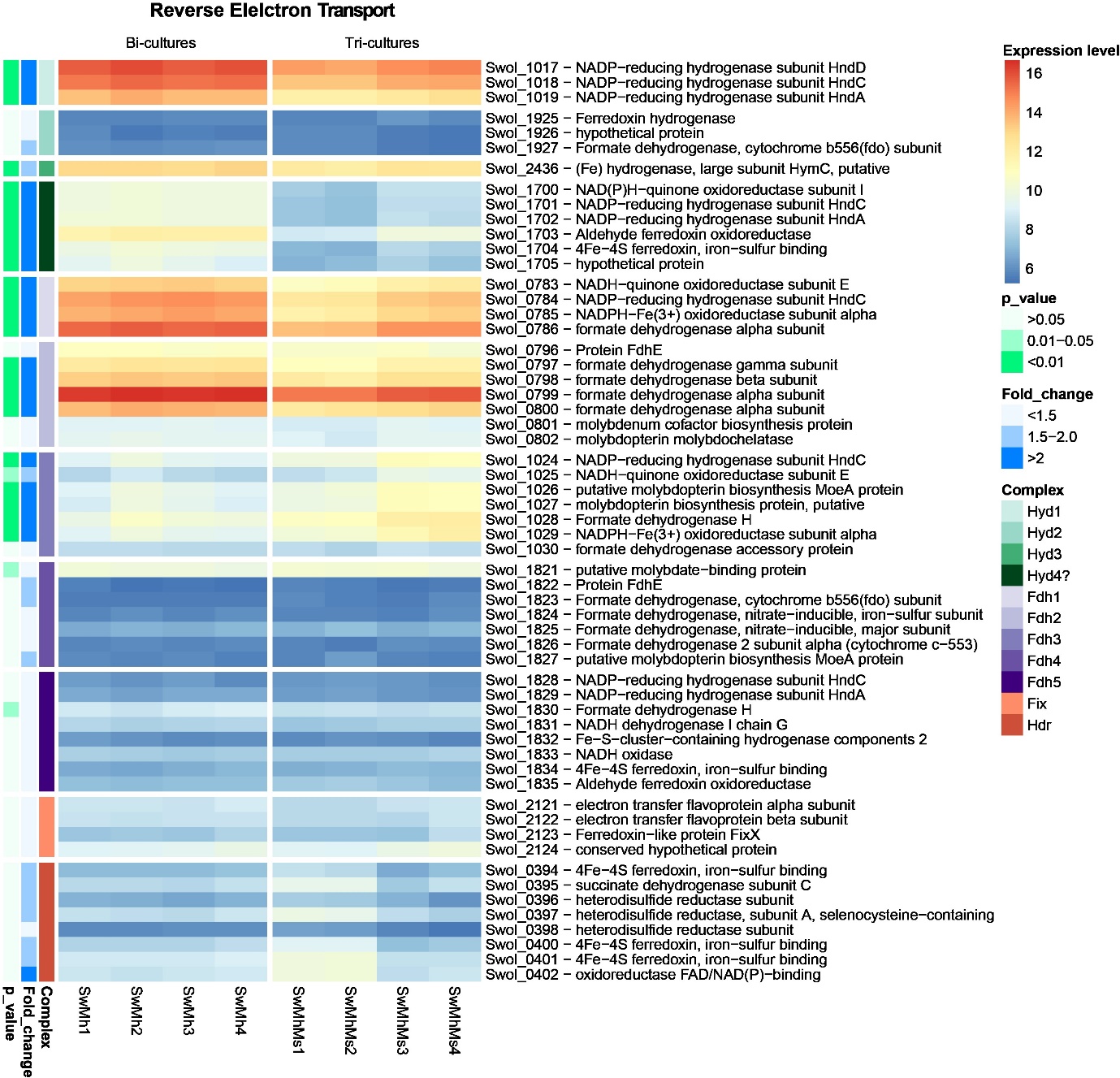


**Figure S6 |** Differential expression of *S. wolfei* genes involved in reverse electron transfer in bi- and tri-cultures. Variance stabilizing transformation on DESeq2 normalized counts was applied for visualization.


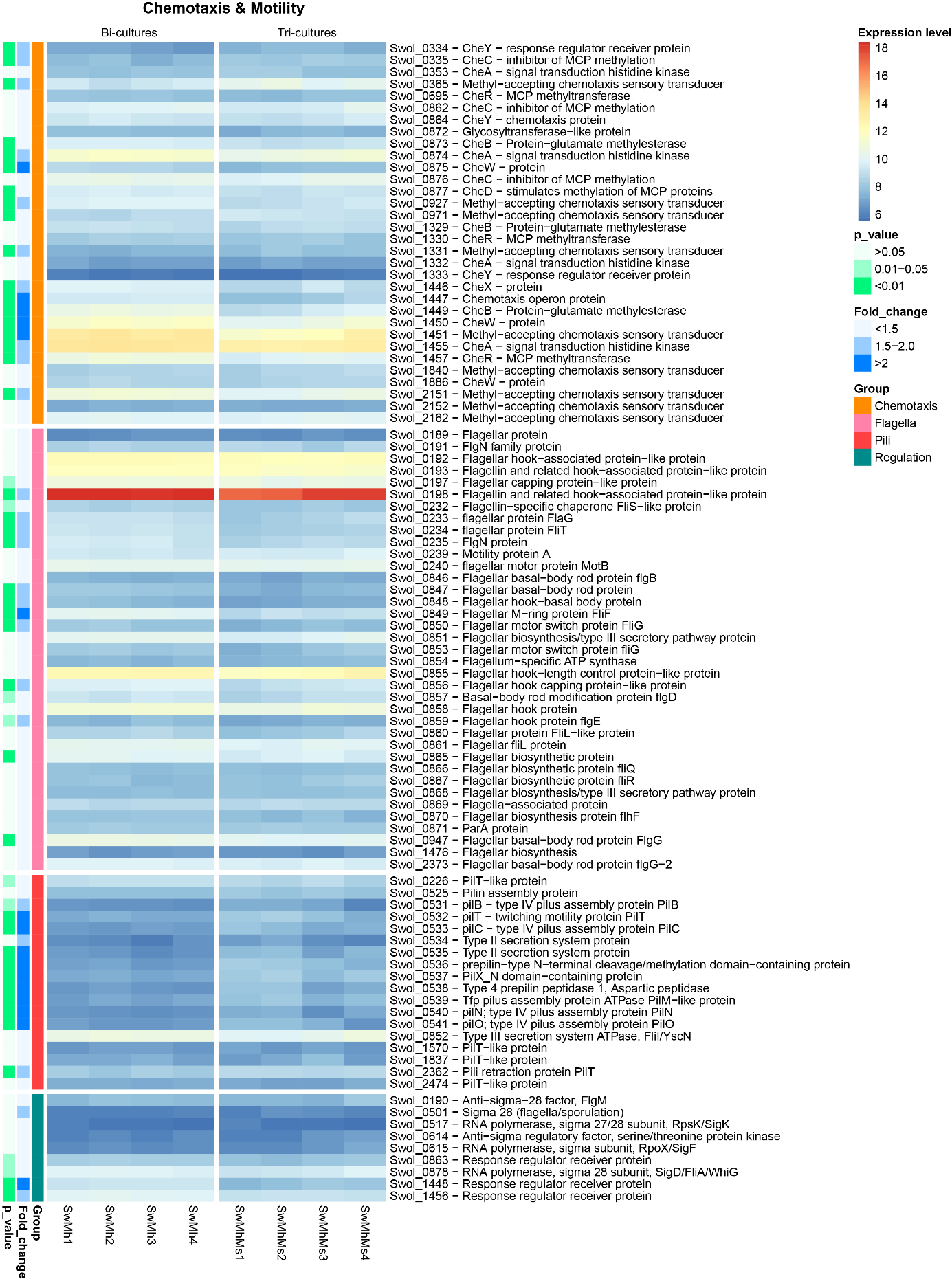


**Figure S7 |** Differential expression of *S. wolfei* genes involved in chemotaxis and motility in bi- and tri-cultures. Variance stabilizing transformation on DESeq2 normalized counts was applied for visualization.


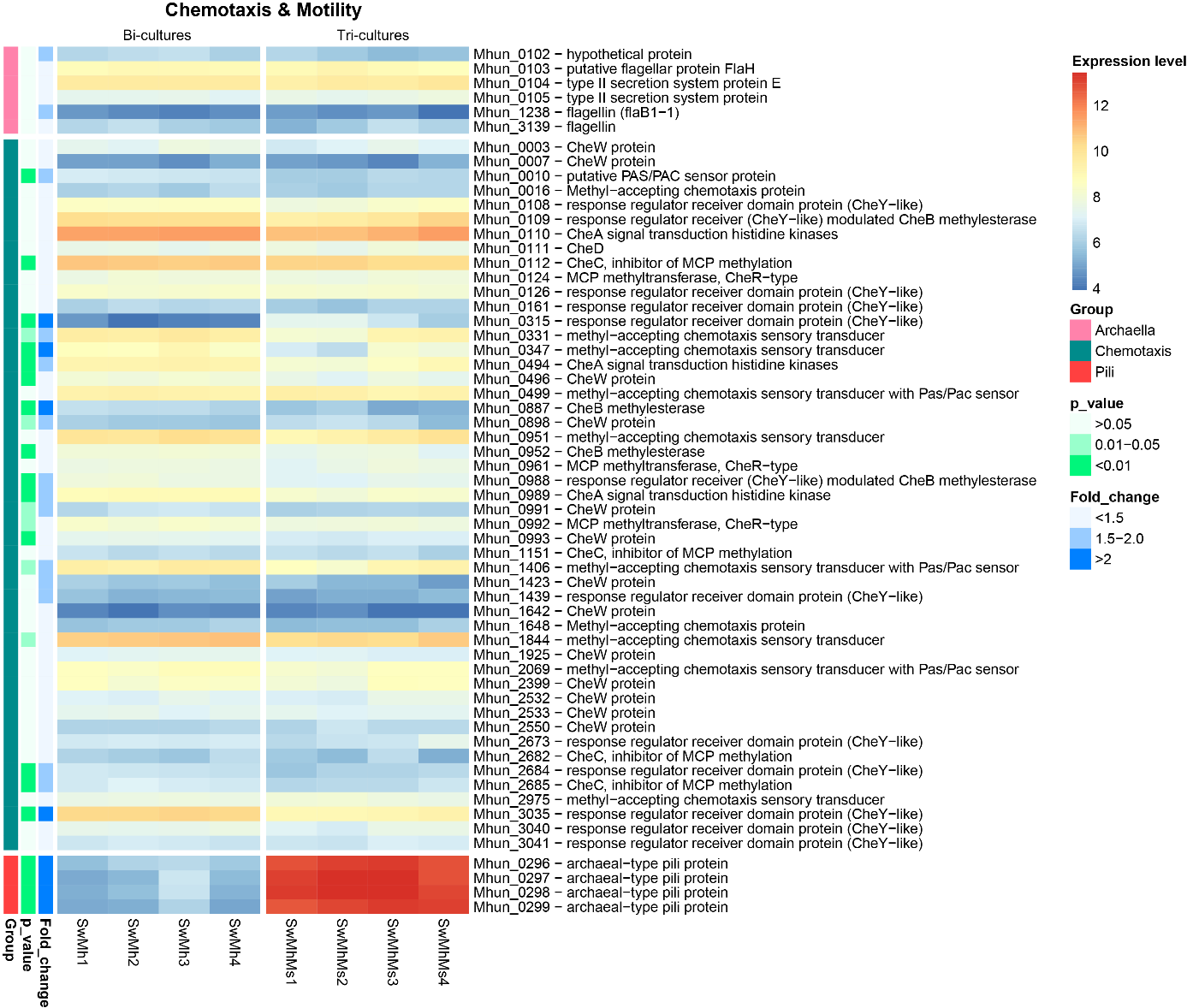


**Figure S8 |** Differential expression of *M. hungatei* genes involved in chemotaxis and motility in bi- and tri-cultures. Variance stabilizing transformation on DESeq2 normalized counts was applied for visualization.
